# Mobility and other predictors of hospitalization for urinary tract infection: a retrospective cohort study

**DOI:** 10.1186/1471-2318-8-31

**Published:** 2008-11-25

**Authors:** Mary AM Rogers, Brant E Fries, Samuel R Kaufman, Lona Mody, Laurence F McMahon, Sanjay Saint

**Affiliations:** 1 Division of General Medicine, Department of Internal Medicine, University of Michigan, Ann Arbor, Michigan, USA; 2 Patient Safety Enhancement Program, Veterans Affairs Medical Center and University of Michigan Health System, Ann Arbor, Michigan, USA; 3 Institute of Gerontology, School of Medicine, University of Michigan, Ann Arbor, Michigan, USA; 4 School of Public Health, University of Michigan, Ann Arbor, Michigan, USA; 5 Geriatric Research, Education, and Clinical Center, Veterans Affairs Ann Arbor Healthcare System, Ann Arbor, Michigan, USA; 6 Division of Geriatric Medicine, Department of Internal Medicine, University of Michigan, Ann Arbor, Michigan, USA; 7 Center for Practice Management and Outcomes Research, Ann Arbor Veterans Affairs Health Services Research & Development Center of Excellence, Ann Arbor, Michigan, USA

## Abstract

**Background:**

Many hospitalizations for residents of skilled nursing facilities are potentially avoidable. Factors that could prevent hospitalization for urinary tract infection (UTI) were investigated, with focus on patient mobility.

**Methods:**

A retrospective cohort study was conducted using 2003–2004 data from the Centers for Medicare and Medicaid Services. The study included 408,192 residents of 4267 skilled nursing facilities in California, Florida, Michigan, New York, and Texas. The patients were followed over time, from admission to the skilled nursing facility to discharge or, for those who were not discharged, for 1 year. Cox proportional hazards regression was conducted with hospitalization for UTI as the outcome.

**Results:**

The ability to walk was associated with a 69% lower rate of hospitalization for UTI. Maintaining or improving walking ability over time reduced the risk of hospitalization for UTI by 39% to 76% for patients with various conditions. For residents with severe mobility problems, such as being in a wheelchair or having a missing limb, maintaining or improving mobility (in bed or when transferring) reduced the risk of hospitalization for UTI by 38% to 80%. Other potentially modifiable predictors included a physician visit at the time of admission to the skilled nursing facility (Hazard Ratio (HR), 0.68), use of an indwelling urinary catheter (HR, 2.78), infection with *Clostridium difficile *or an antibiotic-resistant microorganism (HR, 1.20), and use of 10 or more medications (HR, 1.31). Patient characteristics associated with hospitalization for UTI were advancing age, being Hispanic or African-American, and having diabetes mellitus, renal failure, Parkinson's disease, dementia, or stroke.

**Conclusion:**

Maintaining or improving mobility (walking, transferring between positions, or moving in bed) was associated with a lower risk of hospitalization for UTI. A physician visit at the time of admission to the skilled nursing facility also reduced the risk of hospitalization for UTI.

## Background

Hospital admissions due to pneumonia and infections of the urinary tract or kidney account for the greatest number of potentially avoidable hospitalizations for individuals who reside in skilled nursing facilities (SNF) [1]. In a report of 1.26 million nursing home residents in the United States, the rate of hospitalization was greatest in residents whose primary diagnosis was genitourinary system disease and among these patients, approximately 1 in 3 hospitalizations were for a urinary tract infection (UTI) [2]. Despite the frequency of UTI among residents of SNF, predictors of hospitalization for UTI among this patient population have not been widely studied.

One potential predictor is physical functioning. There has been recent interest in expanding research to evaluate functional status, including measures of mobility, as either risk factors or outcomes for infectious diseases in the elderly [3]. Some reports have indicated immobility as a predisposing factor for UTI and there have been small trials of interventions to improve endurance and strength and to decrease urinary incontinence in residents of SNF [4-7]. Moreover, indwelling urinary catheters, which are known risk factors for UTI, may impede mobility [8]. However, the independent effect, if any, of reduced mobility on UTI has not yet been fully explored. Therefore, we investigated risk factors of hospitalization for UTI with the goal of identifying potentially modifiable factors such as mobility and urinary catheter use.

## Methods

Subjects were all newly-admitted individuals to a SNF in year 2003 in California, Florida, Michigan, New York, and Texas. These five states were selected for their large population size and diverse location across the United States. Only those 65 years of age and older were included. In this retrospective cohort study, the patients were followed from the time of SNF admission to either discharge from the SNF or one year after the admission (for those who were not discharged). A SNF is a facility which primarily provides inpatient skilled nursing care and related services to patients who require medical, nursing, or rehabilitative services but does not provide the level of care or treatment available in a hospital. Skilled nursing facility care is defined as a level of care that requires the daily involvement of skilled nursing or rehabilitation staff such as intravenous injections or physical therapy.

Data were obtained from the 2003 and 2004 Long Term Minimum Data Set (MDS) from the Centers for Medicare and Medicaid Services (CMS). The MDS is a standardized, primary screening and assessment tool of health status which forms the foundation of the comprehensive assessment for all residents of SNFs certified to participate in Medicare or Medicaid. The MDS contains items that measure physical, psychological and psychosocial functioning. Information regarding the first hospitalization during the follow-up period was obtained from the MedPAR files from CMS which contain administrative data regarding inpatient hospital stays, including admission dates and principal diagnosis or reason for hospitalization. The principal diagnoses codes used to define UTI were acute pyelonephritis (590.1), renal and perinephric abscess (590.2), pyeloureteritis cystica (590.3), unspecified pyelonephritis (590.80), unspecified infection of the kidney (590.9), acute cystitis (595.0), or UTI with site not specified (599.0).

Information regarding patient characteristics was obtained from the MDS at two different assessments: (1) the initial admission assessment, which was completed within 14 days of the admission to the SNF; and (2) the last assessment which was completed prior to discharge from the SNF or, for those who were not discharged, the last assessment within the 1-year period of observation. The last assessment may have been a quarterly, change-in-status, or annual assessment.

Demographic information was extracted from the MDS, as well as disease diagnoses at the time of admission to the SNF. Information was also obtained regarding an advanced directive of "do not hospitalize", the number of medications, infection with an antibiotic-resistant organism or *Clostridium difficile*, and UTI within the last 30 days of the assessment. Indwelling urinary catheter use any time within 14 days of the last assessment was obtained. Information regarding physician visits was recorded based on the question, "In the last 14 days (or since admission if less than 14 days in facility) how many days has the physician (or authorized assistant or practitioner) examined the resident?" and was obtained for both the first and last assessments.

Mobility was evaluated using data on the ability to walk in the room or corridor (a) independently, (b) with limited assistance or supervision, or (c) with extensive assistance, total dependence or no walking observed. Improvement in walking was measured as transitioning to a higher degree of walking ability (using the categories above) from the time of the first assessment to the last assessment. To evaluate mobility in patients with existing physical problems such as confinement to a wheelchair or a history of stroke, mobility was ascertained using data on the ability to move positions within the bed or the ability to transfer to and from the bed, chair, wheelchair, or standing position (a) independently, (b) with limited assistance or supervision, or (c) with extensive assistance, total dependence or the activity was not observed. Improvement in transfer mobility was measured as transitioning to a higher degree of bed mobility or transfer (using the categories above) from the first to the last assessment.

Differences in categorical data were assessed using Pearson's chi-squared test, with alpha set at 0.05, two-tailed. Missing data occurred in 0% to 0.3% of the independent variables and were imputed with best-subset regression, using the predicted value of the missing observations from the best available subset of predictor variables in the observed data. Predictors were regressed on time to (first) hospitalization for UTI using Cox proportional hazards regression and the Breslow method for tied failures. Follow-up time commenced at the time of admission to the SNF. Patients who were discharged from the SNF for reasons other than hospitalization for UTI were censored at the time of discharge from the SNF. Patients who were not discharged from the SNF were censored at the time of their last observation within the one year period after admission. Hazard ratios (HR) and 95% confidence intervals were calculated, with the outcome being time to hospitalization for UTI (in days). Predictors were categorized as potentially modifiable preventive factors (HR<1.0), potentially modifiable risk factors (HR>1.0), and patient characteristics. Robust estimates of variance were calculated accounting for clustering within a SNF. The Huber-White sandwich estimator of variance was used to produce standard errors corrected for intragroup (facility) correlation of measures. In addition, to assess the association between change in status over time (i.e., improvement in mobility) and hospitalization for UTI, proportional hazards regression was used for those residents who remained in the SNF long enough to have two assessments (n = 239,399). Two factors that could potentially influence motivation to move (i.e., depression and pain) were considered as possible confounders. The final models included adjustment for depression, pain frequency, and pain intensity at the time of the last assessment. Analyses were performed using Stata/SE 9.2 (StataCorp LP, College Station, TX).

This study received approval by CMS and the human subjects review board at the University of Michigan.

## Results

There were 408,192 elderly individuals who were admitted to 4267 SNFs in California, Florida, Michigan, New York, and Texas in 2003. The majority of patients (55.1%) remained in the SNF for 30 days or less after the initial admission assessment, while 21.4% remained for 31–90 days and 23.5% remained for more than 90 days. The median length of stay in the SNF was 25 days.

The majority of the residents were women (66%), Caucasian (82%), and 80 years of age or older (62%). Most (82%) were initially admitted to the SNF from an acute care hospital.

Of the 408,192 SNF residents, 89,538 (21.9%) were subsequently hospitalized during the observation period. There were 3961 residents who were hospitalized for UTI, yielding an incidence rate of 45.3 persons hospitalized for UTI per 1000 person-years of observation (95% CI, 43.9 to 46.8 per 1000). Of those SNF residents who were hospitalized for UTI, 41.9% occurred within the 30 days after the first SNF assessment, 25.4% occurred within 31–90 days after the first assessment, and 32.7% occurred after 90 days from the first assessment.

Results from the regression model are given in Table 1, with all predictors listed in the table regressed simultaneously. There were three potentially modifiable preventive factors of hospitalization for UTI: walking ability, a physician visit at the time of admission to the SNF, and an advanced directive of "do not hospitalize." Independent walking was associated with a 69% decreased risk of hospitalization for UTI compared to residents who did not walk or required extensive assistance to walk. Being a supervised walker yielded a 45% decreased risk of hospitalization for UTI. Overall, 11.9% of residents were independent walkers at the last assessment and 41.5% of residents required supervision or limited assistance.

**Table 1:** Hazard ratios for predictors of hospitalization for urinary tract infection (n = 408,192).

**Predictors**	**Percent with attribute**	**Hazard Ratio**	**95% Confidence Interval**
***Potentially Modifiable Preventive Factors***			
Walking ability at last assessment:			
Requires extensive assistance or does not walk	46.6%	1.00	reference
Walking with limited assistance or supervision	41.5%	0.55	0.51, 0.60
Independent walker	11.9%	0.31	0.27, 0.36
Physician visit at admission to skilled nursing facility	84.2%	0.68	0.63, 0.74
Advanced directive to "do not hospitalize" at last assessment	2.7%	0.40	0.31, 0.54
***Potentially Modifiable Risk Factors***			
Indwelling urinary catheter at last assessment	15.7%	2.78	2.57, 3.01
Physician visit at last assessment	76.8%	2.04	1.89, 2.19
Urinary tract infection at admission to skilled nursing facility	19.0%	1.57	1.46, 1.69
Ten or more different medications at last assessment	44.8%	1.31	1.23, 1.40
Infection with *C. difficile *or antibiotic-resistant organism at last assessment	4.2%	1.20	1.04, 1.38
***Patient Characteristics***			
Age (years):			
65–69	7.5%	1.00	reference
70–74	11.8%	0.98	0.83, 1.15
75–79	19.0%	1.12	0.96, 1.30
80–84	24.1%	1.27	1.10, 1.47
85–89	21.3%	1.35	1.17, 1.57
≥ 90	16.3%	1.31	1.12, 1.53
Race/Ethnicity:			
White, not of Hispanic origin	81.9%	1.00	reference
Hispanic	7.3%	1.22	1.08, 1.38
African-American, not of Hispanic origin	8.3%	1.12	1.01, 1.25
Asian/Pacific Islander	2.2%	0.83	0.65, 1.06
Native American	0.2%	0.81	0.43, 1.56
Parkinson's disease	5.2%	1.34	1.20, 1.49
Diabetes mellitus	27.4%	1.25	1.16, 1.34
Dementia	31.3%	1.24	1.16, 1.32
Renal failure	7.7%	1.14	1.02, 1.28
Stroke, TIA or hemiplegia	21.6%	1.13	1.05, 1.21

The risk of hospitalization for UTI significantly decreased by 32% for patients who received at least one visit by a physician at the time of admission to the SNF. Overall, 84.2% of patients admitted to a SNF had a physician visit within the first 14 days of admission. When the actual number of physician visits at the time of admission was regressed on hospitalization for UTI, the HR was 0.93 (95% CI, 0.90 to 0.95) indicating a 7% reduction in risk of hospitalization with every physician visit. However, a physician visit at the last assessment was associated with an increased risk of hospitalization for UTI, with a HR of 2.04. In additional analyses, the regression model in Table 1 was rerun so that the hazard ratio for a physician visit at the last assessment reflected only those visits that occurred after the initial admission assessment. This yielded a hazard ratio of 2.24 (95% CI 2.06 to 2.43) for the association between a physician visit at the last assessment and hospitalization for UTI.

Having an advanced directive to "do not hospitalize" at the time of admission did not always preclude hospitalization. Overall, 2.7% of the residents had an advanced directive of "do not hospitalize" at their last assessment. Of the 3961 patients hospitalized for UTI, 60 (1.5%) had such a directive at their last SNF assessment prior to transferring to the hospital. The directive was associated with a 60% reduction in the risk of hospitalization for UTI. When patients with a "do not hospitalize" order (2.7% of the sample) were excluded from the regression model, the hazard ratios for the other predictors, listed in Table 1, did not change.

The risk of hospitalization for UTI was 2.78 times greater in those patients who used an indwelling urinary catheter compared to those who did not use a catheter. An indwelling urinary catheter was present in 15.7% of residents at the last assessment. The presence of a UTI at the time of admission to the SNF increased the risk of hospitalization for UTI by 57%, independent of indwelling catheter use. In addition, there was a 20% increased risk of hospitalization for UTI for patients who were infected with either *C. difficile *or an antibiotic-resistant microorganism at the last assessment. The prevalence of infection due to an antibiotic-resistant organism was 2.7% and due to *C. difficile *was 1.6% at the last assessment in the SNF.

Patient characteristics associated with hospitalization for UTI included advancing age, Parkinson's disease, diabetes mellitus, dementia, renal failure, and stroke, transient ischemic attack (TIA) or hemiplegia. Race and ethnicity were also related to hospitalization for UTI; both Hispanics and African-Americans had an elevated risk of hospitalization compared to whites not of Hispanic origin.

All the predictors listed in Table 1 remained statistically significant after further adjustment for gender, body mass index, depression, arteriosclerotic heart disease, congestive heart failure, deep vein thrombosis, peripheral vascular disease, emphysema or chronic obstructive pulmonary disease, arthritis, hip fracture, osteoporosis, pathogenic bone fracture, missing limb, cancer, multiple sclerosis, comatose status, brain injury, paraplegia, quadriplegia at admission, and pain frequency and intensity at the last assessment. Of these additional variables added to the model, only two were statistically significant, gender and comatose state. Women were more likely to be hospitalized for UTI than men (HR, 1.08; 95% CI, 1.00 to 1.16; p = 0.043). Patients in a comatose state were less likely to be hospitalized for UTI than those not in a coma (HR, 0.35; 95% CI, 0.16 to 0.79; p = 0.011).

A secondary analysis was completed for those in whom a UTI developed during the SNF stay, which compared patients hospitalized for UTI versus those not hospitalized for UTI. Significant predictors of hospitalization for UTI were walking ability (HR = 0.40 for independent walking), indwelling catheter (HR = 2.66), a physician visit at admission to the SNF (HR = 0.66), a physician visit at the last assessment (HR = 1.83), African-American race (HR = 1.50), diabetes (HR = 1.51), and stroke (HR = 1.38).

Given the variability in length of SNF stay, the regression model was rerun for only those patients who stayed within the SNF for more than 90 days (n = 95,861). The predictors of hospitalization for UTI remained the same as those shown in Table 1 with the following exceptions: advancing age, race, infection with *C. difficile *or an antibiotic-resistant microorganism, renal failure, stroke, and Parkinson's disease were no longer statistically significant at the 0.05 level.

The relation between walking ability and hospitalization for UTI was examined further in patients who remained within the SNF long enough to receive two assessments (n = 239,399), so that improvement over time could be evaluated. Of the SNF residents hospitalized for a UTI, 69% had two assessments. The HRs for the maintenance of independent walking or improvement in walking are listed in Table 2. There was a 39% to 76% reduction in the risk of hospitalization for UTI across patients with various types of disease conditions such as congestive heart failure, osteoporosis, arthritis, and stroke. All the HRs in Table 2 were adjusted for the significant predictors as listed in Table 1, as well as depression, pain frequency and pain intensity at the last assessment.

**Table 2:** Hazard ratios for the association between maintaining or improving the ability to walk over time and hospitalization for urinary tract infection, by disease.^1^

**Analyses Restricted to Residents with:**	**Number**	**Percent that maintained or improved walking ability**	**Hazard Ratio**	**95% Confidence Interval**
Depression	65,951	29.0%	0.50	0.41, 0.61
Dementia	85,500	27.7%	0.46	0.39, 0.54
Hip fracture	25,840	31.3%	0.24	0.16, 0.36
Osteoporosis	38,538	30.1%	0.42	0.32, 0.55
Arthritis	63,129	30.0%	0.45	0.37, 0.56
Parkinson's disease	13,828	24.0%	0.48	0.32, 0.72
Stroke, transient ischemic attack, or hemiplegia	54,904	22.8%	0.49	0.40, 0.61
Congestive heart failure	54,837	27.0%	0.55	0.45, 0.68
Peripheral vascular disease	22,384	25.0%	0.48	0.33, 0.68
Chronic obstructive pulmonary disease	44,694	29.9%	0.51	0.39, 0.67
Diabetes mellitus	64,871	27.5%	0.47	0.39, 0.56
Cancer	24,764	27.6%	0.61	0.44, 0.85
All residents, regardless of disease	239,399	29.4%	0.47	0.42, 0.52

1. Only patients with assessments of walking ability during two time periods were included. Patients with paraplegia, quadriplegia, or comatose condition were excluded.

We also examined whether an improvement in mobility was related to hospitalization for UTI in those individuals with considerable impediments to mobility, such as those in wheelchairs or bedfast. Table 3 lists the HRs for improvement in the ability to move within the bed or when transferring to and from the bed or chair from first to last assessments. For those patients who used wheelchairs as their primary mode of locomotion at the time of SNF admission, an improvement in the ability to move in bed or transfer decreased the risk of hospitalization for UTI by 38%. Likewise, patients who were bedfast, had missing limbs, or with a previous history of stroke or hip fracture had a significant reduction in the risk of hospitalization for UTI. There was no statistically significant association between improvement in mobility and hospitalization for UTI for residents with multiple sclerosis or for those with paraplegia. All HRs in Table 3 were adjusted for the predictors of hospitalization for UTI as listed in Table 1, as well as for depression, pain frequency, and pain intensity at the last assessment.

**Table 3:** Hazard ratios for the association between improvement in bed mobility or transferring positions and hospitalization for urinary tract infection, by patient characteristics.^1^

**Characteristic at Time of SNF Admission**	**Number**	**Percent that improved mobility**	**Hazard Ratio**	**95% Confidence Interval**
Wheelchair primary mode of locomotion	167,146	27.0%	0.62	0.56, 0.70
Bedfast all or most of time	9,380	14.2%	0.43	0.22, 0.81
Missing limb	4,502	22.3%	0.20	0.06, 0.64
Stroke, TIA, or hemiplegia	55,032	21.8%	0.58	0.47, 0.72
Hip fracture	25,854	35.9%	0.46	0.34, 0.62
Multiple sclerosis	607	17.3%	0.46	0.02, 12.14
Paraplegia	625	15.2%	1.05	0.24, 4.64

1. Only patients with assessments of mobility during two time periods were included. Patients with quadriplegia or comatose condition were excluded.

For analyses reported in Tables 1, 2 and 3, we conducted a sensitivity analyses matching on the time of the assessments. The protective associations between mobility measures and hospitalization for UTI remained for the results included in Tables 1 and 2. For Table 3, the sensitivity analyses did not reveal any effect modification across assessment times, with the exception of subjects whose primary mode of locomotion was a wheelchair. In these patients, the degree of improvement in mobility was more strongly protective of hospitalization for UTI for those with shorter length of stay (median, 21 days) than those with longer length of stay in the SNF (median, 143 days). The hazard ratio was 0.44 (95% CI, 0.34 to 0.58) in those with shorter length of stay and was 0.83 (95% CI, 0.73 to 0.95) in those with longer length of stay. The interaction term was statistically significant (p < 0.001).

## Discussion

The results suggest that mobility afforded the strongest protection against hospitalization for UTI, independent of the presence of an indwelling urinary catheter, age, and the presence of various comorbidities. Regardless of whether the patients was admitted to the SNF with a previous history of a stroke, coronary heart disease, dementia, hip fracture, depression or other common conditions in the elderly, the ability to walk considerably reduced the risk of hospitalization for UTI. There was a 69% reduction in the risk of hospitalization for UTI for independent walkers and a 45% reduction in those who required limited assistance or supervision with walking. Furthermore, improvement in walking ability over time or maintenance of the ability to walk was observed in 29% of residents, which reduced the risk of hospitalization for UTI by 53%. For some patients with severely limited mobility at the time of admission, increasing the ability to move in bed or to transfer oneself also decreased the risk of hospitalization for UTI. It was particularly protective in those residents with missing limbs, who had an 80% reduced risk of hospitalization for UTI with improved mobility. It was also protective in those residents who were bedfast at the time of admission to the SNF and in those whose primary mode of locomotion was a wheelchair. For those patients using wheelchairs, more rapid improvement in mobility over time yielded a greater reduction in the risk of hospitalization for UTI than improvement in mobility over a longer period of time.

Exercise is perhaps under-appreciated in its ability for proper functioning of the urinary tract. There are several possible underlying mechanisms for this association. The first may be through a reduction in long periods of urinary stasis which have been shown to increase the risk of UTI [9]. Experimental studies have shown that urinary flow reduces the numbers of *E. coli*, *S. aureus*, and *P. mirabilis *in the bladder by more than 99.9% [10]. Another mechanism by which mobility may influence the risk of severe UTI could be through the enhanced abilities of the resident to address issues regarding voiding and to prevent or better manage urinary incontinence. In a randomized controlled trial of an intervention to increase mobility and functional ability in residents of nursing homes, the frequency of urinary incontinence significantly decreased and the walking ability of the residents increased [4]. Ouslander and colleagues found similar improvements in endurance, strength, and urinary incontinence with mobility exercises in residents of VA nursing homes, with 67% of participants in the program showing either maintenance or improvement [5]. A reduction in incontinence was also found in a randomized trial of mobility training in elderly women [6]. Indeed, various approaches to enhance mobility are already utilized in some nursing homes, but for other reasons [11,12].

There are several limitations of using retrospective data to study these hypotheses. This investigation considered only the most serious of UTI episodes – those requiring hospitalization. Infections of the urinary tract may be difficult to detect in a SNF since symptomatic UTI requires the recognition of multiple signs and symptoms, some of which include laboratory testing and physician involvement in diagnosis or treatment. Therefore, this study was designed to investigate only UTIs that were severe enough to warrant a hospitalization. Another limitation is that immobility could be an indicator of frailty or the presence of multiple comorbidities. However, the hazard ratios for walking and improvement in walking remained significant after adjustment for multiple conditions. Furthermore, when analyses were restricted to individuals with severe existing mobility problems (those who were bedfast, in a wheelchair, or with a missing limb), improvement in the ability to move in bed or transfer positions did significantly reduce the likelihood of hospitalization. Moreover, adjustment was made for conditions that may reduce motivation to move, such as depression, pain frequency, pain intensity, and the presence of an indwelling catheter, which has been termed a "one-point restraint" [8]. Nevertheless, there may be other elements of functional capacity which were not measured in this study, for which a prospective approach or an intervention could more directly answer.

Another limitation is the inability to directly evaluate accuracy of the mobility measures utilized in this study. Graney and Engle assessed reliability of mobility measures on the MDS and found no significant within-subject differences in means for bed mobility, transferring, walking in corridor, or walking in the room (p values from 0.305 to 0.889) [13]. Bates-Jensen and colleagues found significant agreement for independent bed mobility (kappa = 0.639; p = 0.021) when assessments were made within 30 days of each other [14]. They also found good agreement in MDS bed-mobility levels of needing extensive assistance and total dependence (86%, 90%, respectively); such levels were similar to those used in our study here. Landi and colleagues found significant agreement (r^2 ^= 0.74; p < 0.001) between the Barthel Index (which includes mobility measures) and corresponding MDS measures [15]. Byers found a significant correlation between the physical ability component of the Functional Independence Measure and the corresponding mobility measures in the MDS (r = 0.822, p < 0.01) [16].

Another protective factor for hospitalization was a visit by a physician at the time of admission to the SNF; conversely, a physician visit at the last assessment increased the risk of hospitalization. It is likely that a physician visit prior to SNF discharge was indicative of problems that may have occurred during the SNF stay which warranted a medical opinion and therefore, were associated with transfer to a hospital. A physician visit, however, at the time of admission to the SNF reduced hospitalization for UTI by 32%. Although the reasons for this could not be directly assessed in these data, a survey by the American Medical Association indicated that most physicians who practiced in nursing homes spent less than two hours each week caring for their nursing home patients [17]. Lack of familiarity with SNF residents was cited by medical directors and directors of nursing as a reason for overhospitalization [18]. The ability to obtain an evaluation of a patient in less than 4 hours by an on-site doctor or nurse practitioner was the most difficult issue in resource availability for decisions to hospitalize nursing home residents [18].

There have been few previous studies of predictors for hospitalization for UTI. Wald and colleagues recently reported an increased likelihood of rehospitalization for UTI with extended use of indwelling urinary catheters in surgical patients who had been admitted to a SNF postoperatively from an acute care hospital [19]. Levy and colleagues found that nonwhite (African-American and Latinos) were more likely to be hospitalized or be assessed by a practitioner for UTI than whites – similar to the results from our study [20]. This association between race/ethnicity and hospitalization remained after adjustment for other known predictors in our investigation and therefore, could not be explained by racial differences in the presence of diabetes mellitus or differential use of advanced directives. Other predictors of hospitalization for UTI found in our study have previously been shown to be related to either UTI (indwelling urinary catheter, previous UTI, medication use) or hospitalization (age, infection with *C. difficile *or antibiotic-resistant organism) [21,22].

## Conclusion

In conclusion, the results suggest that the benefits of mobility may be extended to those elderly who are often considered to be the most disabled in our society. Despite the presence of severe conditions that compromise mobility, an improvement in mobility may be beneficial in preventing hospitalization for UTI, even when it is just within the bed or from bed to chair.

## Competing interests

Dr. Saint has received honoraria in the past for lecturing on catheter-related urinary tract infection from the VHA (a group purchasing organization) and CR Bard (a catheter manufacturer). The remaining authors declare that they have no competing interests.

## Authors' contributions

MAMR was involved in study concept and design, acquisition of the data, data analyses, interpretation of data, critical review of the manuscript, and manuscript preparation. BEF was involved with study concept and design, acquisition of the data, interpretation of data, and critical review of the manuscript. SRK was involved in study concept and design, acquisition of the data, data analyses, interpretation of data, and critical review of the manuscript. LM was involved in study design, interpretation of data, and manuscript preparation, and critical review of the manuscript. LFM Jr. was involved with study concept and design, acquisition of the data, interpretation of data, and critical review of the manuscript. SS was involved with study concept and design, acquisition of the data, interpretation of data, and critical review of the manuscript.

## Pre-publication history

The pre-publication history for this paper can be accessed here:


